# Diurnal Expression of the Per2 Gene and Protein in the Lateral Habenular Nucleus

**DOI:** 10.3390/ijms160816740

**Published:** 2015-07-23

**Authors:** Zhigong Zhao, Haiyan Xu, Yongmao Liu, Li Mu, Jinyu Xiao, Hua Zhao

**Affiliations:** Department of Physiology, College of Basic Medical Sciences, Jilin University, 126 Xinmin Street, Changchun 130021, China; E-Mails: studayhard518@sina.com (Z.Z.); hyxu@jlu.edu.cn (H.X.); lymmm8338@sina.com (Y.L.); muli_xf@126.com (L.M.); zacshaw@outlook.com (J.X.)

**Keywords:** period2 gene, circadian rhythm, lateral habenular nucleus, suprachiasmatic nucleus, running-wheel activity

## Abstract

The suprachiasmatic nucleus plays an important role in generating circadian rhythms in mammals. The lateral habenular nucleus (LHb) is closely linked to this structure. Interestingly, the LHb shows a rhythmic firing rate *in vivo* and *in vitro*, and sustained oscillation of rhythmic genes *in vitro*. However, under the *in vivo* condition, whether rhythmic gene expression in the LHb has circadian rhythms remains unknown. In this study, we examined LHb tissue in rats to determine Period2 (Per2) gene and protein expression at six zeitgeber time points (ZT2, ZT6, ZT10, ZT14, ZT18, and ZT22) in a 12-h light and 12-h dark (LD) environment. We found that in the LD environment, *Per2* gene expression and PER2 protein levels in the LHb were higher in the day and lower in the night, showing periodic oscillation, with a peak at ZT10 and a trough at ZT22 (*Per2* mRNA) and ZT18 (PER2 protein). We conclude that *Per2* expression and PER2 protein levels in the LHb have rhythmic oscillation *in vivo.* This study provides a basis for further study on the role of the LHb in the circadian rhythm system.

## 1. Introduction

Circadian rhythms are observed in all physiological and behavioral processes of mammals. The suprachiasmatic nucleus (SCN) of the anterior hypothalamus is the major pacemaker of the system for generating and sustaining daily circadian rhythms in mammals [1,2,3]. The daily physiological and behavioral circadian rhythms in rats disappear after the SCN is lesioned bilaterally [1,4]. The SCN generates internal circadian rhythms in many behavioral and physiological functions, and also synchronizes them to environmental light-dark cycles via the direct retinohypothalamic tract [5,6,7]. Therefore, many researchers have focused on the role of the SCN in investigating the regulatory mechanisms of circadian rhythms. However, several studies have raised the possibility that some circadian oscillators of varying strength exist in other brain areas in addition to the SCN [8,9,10]. This indicates that circadian processes in the brain are not solely influenced by the SCN. These nuclei may play an important role in linking SCN output to the system *in vivo*. Therefore, such extra-SCN sites need to be identified to reveal the mechanisms of circadian rhythms.

One of the structures with rhythmicity is the habenular nucleus (Hb), particularly the lateral habenular nucleus (LHb), which plays an important role in conveying limbic forebrain input to mid-brain structures [11,12,13]. The LHb is involved in a wide range of functions, such as stress responses, reproductive behavior, and sleep, which are regulated on a circadian basis [14,15,16]. Some of the affective disorder diseases, which are attributed to the LHb, are related to disrupted circadian rhythms [17,18]. Recently, the LHb structure was suggested as a component of the brain’s circadian system [19,20]. The roles of the LHb in physiological function and disease may be associated with its own rhythmic activities and changes, and may convey the information from the SCN to systems controlling biological circadian rhythms [9,21].

Our previous study showed that the firing rates of rat Hb neurons were altered in response to retinal illumination *in vivo.* In addition, LHb neuronal activity showed rhythmic oscillation in *in vivo* and *in vitro* experiments [22]. Tavakoli-Nezhad reported that the expression of c-Fos protein in the medial division of the LHb showed a rhythmic oscillation in hamsters [23]. Recent research has shown that the transecting fasciculus retroflexus, which is the major efferent pathway of the LHb, alters the daily amount of motor activity and extends the circadian rest–activity rhythm cycle set by the SCN [20]. Circadian oscillators of clock gene expression are present in the LHb *in vitro* [24]. In addition, the LHb receives a vasopressin-containing projection from the SCN [25]. Taken together, these findings suggest that the LHb may be associated with the circadian rhythm system as a downstream structure of the SCN. However, whether rhythmic clock gene/protein expression exists in the LHb *in vivo* and whether it shows zeitgeber time-keeping properties in this structure are unknown.

To address these issues, this study aimed to investigate Period2 (*Per2*) gene expression and PER2 protein levels under 12-h light and 12-h dark (LD) conditions at different time points to identify the diurnal oscillation of LHb activity *in vivo* in rats.

## 2. Results and Discussion

### 2.1. Expression of Per2 mRNA in the LHb of LD Rats

**Figure 1:**
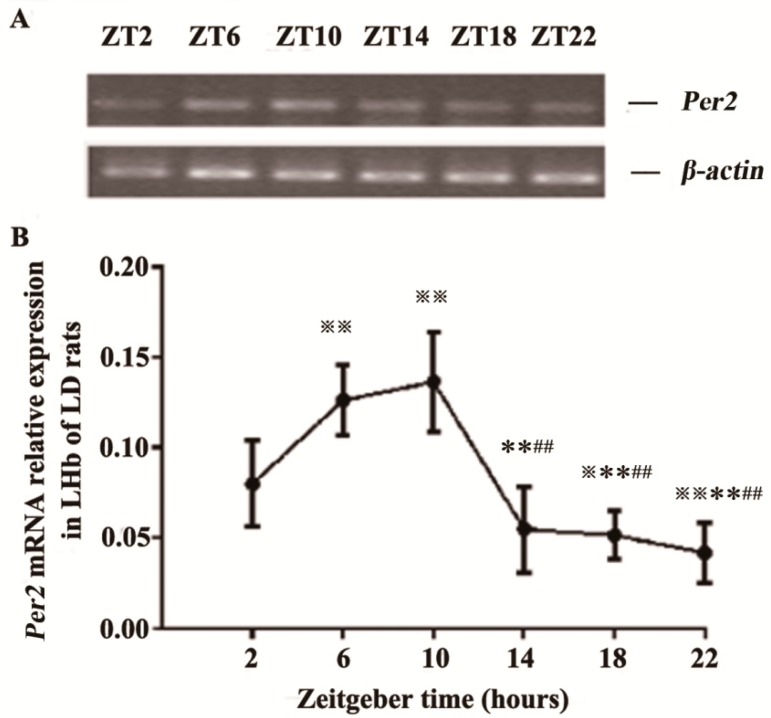
Rhythmic expression of *Per2* mRNA in the lateral habenular nucleus (LHb) of LD rats. (**A**) Representative gel electrophoresis of real-time polymerase chain reaction products stained with ethidium bromide (EB), showing *Per2* and β*-actin* mRNA in the LHb at six ZT time points; and (**B**) Relative quantitative analysis of *Per2* mRNA in the LHb at six ZT time points. All values are normalized against the housekeeping gene β*-actin*. Data are presented as mean ± SD (*n* = 6). ^※^ *p* < 0.05 and ^※※^ *p* < 0.01 compared with ZT2 time point; ** *p* < 0.01, compared with ZT6 time point; ^##^ *p* < 0.01, compared with ZT10 time point.

We determined six zeitgeber time points (ZT2, ZT6, ZT10, ZT14, ZT18, ZT22) in a LD environment to examine diurnal *Per2* mRNA expression in the LHb. Multigroup data in the time series showed significant differences by one-way analysis of variance (ANOVA) (F[5,30] = 21.659, *p* < 0.01). The post hoc analysis was then conducted to compare the differences of every two time points. We found that *Per2* expression peaked at ZT10 and was lowest at ZT22. *Per2* expression at ZT6 was also high, similar to that at ZT10. *Per2* expression at ZT6 and ZT10 was significantly higher than that at ZT2, ZT14, ZT18 and ZT22 (all at *p* < 0.01). *Per2* expression at ZT2 was significantly higher than that at ZT18 (*p* < 0.05) and ZT22 (*p* < 0.01) (Figure 1).

### 2.2. Expression of PER2 Protein in the LHb of LD Rats

**Figure 2:**
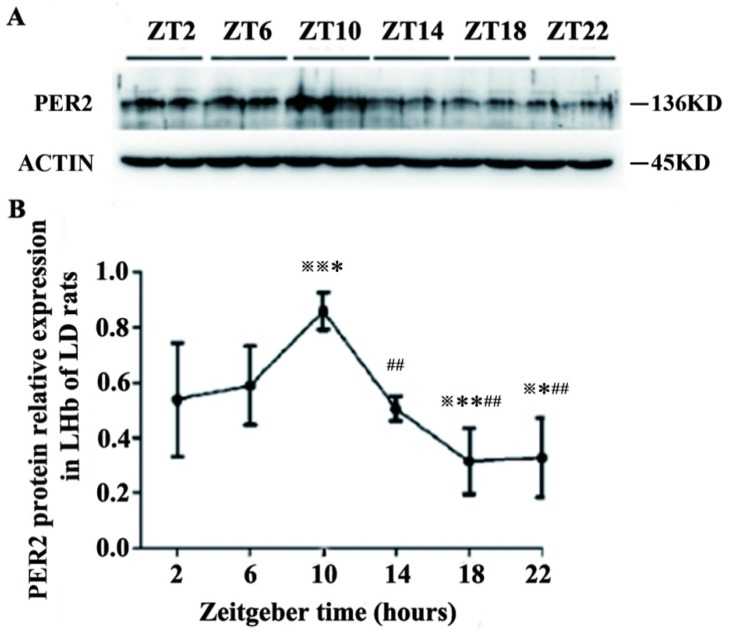
Rhythmic expression of PER2 protein in the LHb of LD rats. (**A**) Western blotting of PER2 and β-actin bands in the LHb at six ZT time points; and (**B**) Relative density of PER2 bands was normalized against β-actin bands at six ZT time points. Data are presented as mean ± SD (*n* = 6). ^※^ *p* < 0.05 and ^※※^ *p* < 0.01 compared with ZT2 time point; * *p* < 0.05 and ** *p* < 0.01 compared with ZT6 time point; ^##^ *p* < 0.01 compared with ZT10 time point.

PER2 relative protein expression at six zeitgeber time points was determined. There was significant differences in multigroup data of the time series by one-way ANOVA (F[5,18] = 9.126, *p* < 0.01). Post hoc comparisons of different time points showed that PER2 expression at ZT10 was significantly higher than that at ZT6 (*p* < 0.05), ZT2, ZT14, ZT18 and ZT22 (*p* < 0.01). PER2 expression at ZT2 was significantly higher than that at ZT18 and ZT22 (*p* < 0.05). The expression at ZT6 was significantly higher than that at ZT18 (*p* < 0.01) and ZT22 (*p* < 0.05). PER2 expression at ZT10 reached a peak, and became the lowest level at ZT18 (Figure 2).

### 2.3. Discussion

The molecular mechanism of circadian rhythm formation depends on the feedback loop of auto-excitation oscillation from biological clock genes and their protein production [26,27]. Among them, Per2 plays an important role in the circadian rhythmic system [28,29,30]. In the current study, Per2 expression at the mRNA and protein levels exhibited rhythmic oscillations, with high expression in the light phase and low expression in the dark phase in the LHb under LD conditions. Per2 expression in the LHb reached a peak at ZT10 and a trough at ZT22 (*Per2* mRNA) and ZT18 (PER2 protein). *Per2* expression at ZT6 was also similar to that at ZT10. These results are consistent with the rhythmicity of LHb neuronal activity that was observed in our previous electrophysiological recordings, which showed that the firing rates of LHb neurons were higher during the day than during the night [22]. Sakhi’s research also showed that the firing rates in LHb brain slices was lower in the early day and higher later in the day [31].These results suggest that there might be functional relevance between circadian oscillations of Per2 gene/protein expression at the molecular level and oscillations in the firing rate at the cellular level in the LHb.

The SCN of the hypothalamus contains a circadian pacemaker that regulates many circadian rhythms in mammals. Electrical activity of SCN neurons and Per2 mRNA/protein expression in the SCN have a circadian rhythm, which is high during the day and low at night [32,33,34]. Rhythmic activity in LHb neurons at cellular and molecular levels coincide with that in the SCN, suggesting that LHb activity might be controlled by the SCN. Similarly, Guilding showed that the peak phase of Per2 expression was not correlated with zeitgeber time in the LHb mouse brain *in vitro* when they used video microscopy imaging and photon-counting of a Per2: luciferase fusion protein [24]. This result indicated that the LHb cannot maintain stabilization of the oscillation phase and synchronization of neuronal activity *in vitro,* when they are devoid of any input from the SCN or other known circadian oscillators. The major output from the SCN includes projections of vasoactive intestinal peptide and arginine vasopressin neurons [35]. Interestingly, the LHb receives a vasopressin-containing projection directly from the SCN, and this plays an important role in conveying circadian information [25,36]. Therefore, rhythmic expression of Per2 gene/protein in the LHb *in vivo* might be regulated by the vasopressin pathway from SCN to LHb to maintain synchronization with SCN rhythms. Further evidence has shown that the SCN has remarkable asymmetry of c-Fos expression in “splitting” hamsters that are maintained in constant illumination. An obvious left-right asymmetry of c-Fos expression was also found in the medial division of the LHb during the active phase [23]. In addition, the SCN and LHb receive a direct retinal projection that conveys light information and responds to light illumination input to induce rhythmic gene expression [22,37,38,39]. This would explain why rhythmic expression of Per2 gene/protein in the LHb is synchronized with that of the SCN *in vivo*.

Many physiological functions that are controlled by the LHb show circadian rhythmicity and the LHb has been shown to generate firing rate rhythms *in vivo* and *in vitro*. These findings at the molecular level demonstrate that the LHb *in vivo* has diurnal expression of Per2 gene/protein in the LD condition. However, how the LHb plays a role in the circadian rhythm system needs to be further investigated.

## 3. Experimental Section

### 3.1. Animals

Male Wistar rats (100–120 g) were provided by the Center for Experimental Animals, Jilin University, Changchun, China (Certification No. SCXK(Ji)2007-0003). Rats were housed in a standard cage with food and water available *ad libitum*, and kept under a standard condition (temperature, 23 ± 2 °C, 12-h light/12-h dark cycles, lights on at 7:00 a.m.) for 1 week. Rats were then transferred into individual cages equipped with a 30-cm diameter running wheel for 3 weeks. The light phase intensity was 400–500 lux and was 35 cm above the cage-top level provided by 8 Watt white fluorescent tubes. There was no light in the room during the dark phase.

Wheel-running activity was recorded using ClockLab data collection and analysis software (Coulbourn Instruments, Actimetrics, Allentown, PA, USA). According to the running-wheel activity only rats that had a good ability of entrainment were chosen for sample collection. All experiments were conducted in accordance with international standards of animal welfare, and were approved by the Institutional Committee for Animal Care Research of Jilin University, Changchun, China.

In LD conditions, the time point of ZT12 was when the lights were turned off and the time point of ZT0 was when the lights were turned on. Rats were anesthetized at six different time points in LD conditions (ZT2, ZT6, ZT10, ZT14, ZT18, and ZT22). The brain was immediately removed and then transferred to ice-cold artificial cerebrospinal fluid at pH 7.4. Two 500 μm thick coronal slices of the lateral habenula were collected with vibration cutting machine (ZQP-86, Shanghai five phase instrument Co., Ltd., Shanghai, China) from Bregma −3.12–4.20 mm. The boundary between medial and lateral habenula is visualized and its distance to midline changes slightly about 10–80 μm along habenular rostrocaudal axis. Because the area from the boundary to midline (medial habenula area) is larger in rostral and caudal side of habenular rostrocaudal axis than middle part of that, we dissected lateral habenula with a thin needle from the rostral and caudal side respevtively to avoid the pollution of the medial habenular tissue*.* The samples were immediately stored at −80 °C until total RNA and protein could be extracted for real-time PCR and western blot experiments. All operations were performed under red light with 8–10 lux during the dark phase to avoid the effect of light.

### 3.2. Detection of Real-Time PCR

Total RNA from LHb tissue was extracted using TRIZOL reagent according to the manufacturer’s protocol. A total of 1 μg RNA was then reverse transcripted to cDNA for quantification of real-time PCR. Real-time-PCR was performed using an SDS 7300 system real-time PCR instrument using Transtart™ Green qPCR Super Mix (Beijing Transgen Biotech Limited Corporation, Beijing, China) with a total reaction volume of 20 μL in accordance with the manufacturer’s instructions. Thermocycling parameters were 95 °C for 30 s, followed by 40 cycles of 95 °C for 5 s, 55 °C for 15 s, and 72 °C for 30 s. The primers were as follows: *Per2* gene sense primer, AGC AAC ACC ACC TTT CAC AA and antisense primer, CGT AGG CTT AGA CCA CCA TC; and β*-actin* gene sense primer, CGT AAA GAC CTC TAT GCC AAC A and antisense primer, TAG GAG CCA GGG CAG TAA TC. SDS 7300 system software was used for recording and analysis of real-time PCR data. According to the *C*_t_ values of *Per2* and β*-actin* of each sample, we analyzed the *Per2* relative content of each sample with the 2^−ΔΔ*C*t^ method.

### 3.3. Western Blotting Experiments

LHb tissues were dissected and total protein of LHb tissues was extracted with radio immunoprecipitation assay (RIPA) containing Phenylmethanesulfonyl fluoride (PMSF). Protein concentrations were determined with BCA protein assay kits. Equal amounts of protein (10 μg for each sample) were electrophoresed on 8% sodium dodecyl sulfate polyacrylamide gel electrophoresis and transferred to a polyvinylidene difluoride membrane with constant voltage of 15 V for 25 min. Nonspecific reactivity was blocked in 5% nonfat dry milk in TBST for 2 h at room temperature. The membrane was then incubated with primary antibodies of Per2 rabbit anti-rat polyclonal antibody (1:1000, Millipore Biological Corporation, Billerica, MA, USA) and β-actin rabbit anti-rat antibody (1:1000, Cell Signaling Technology, Danvers, MA, USA) overnight at 4 °C. After washing with 1× TBST for 5 min three times, the membrane was incubated with HRP-linked goat anti-rabbit IgG antibody (1:1000, Cell Signaling Technology) for 2 h at room temperature. After 5 min of washing the membrane with 1× TBST three times, ECL luminescence liquid (Beijing Apply Gene Technology Company, Beijing, China) was added to the membrane’s surface to detect the target bands. Finally, the membrane was placed in a luminescent imaging machine for photographs (Gene Gnome; Sygene Bio Imaging, Cambridge, UK) and the target bands were analyzed with a gel imaging system (Tanon Gis-2008, Shanghai, China).

### 3.4. Statistical Analysis

Statistical analyses were conducted with SPSS 17.0 software (SPSS Inc., Chicago, IL, USA). Results are expressed as mean ± SD. Multigroup data were analyzed by using one-way ANOVA. The post hoc analysis was then conducted to compare the differences of every two time points using Fisher’s least significant differences (LSD). Probability values <0.05 were considered statistically significant.

## 4. Conclusions

Our study shows that Per2 expression at the mRNA and protein levels in the LHb under LD conditions has diurnal rhythmic oscillations, with high expression during the day and low expression during the night. Our findings will provide a basis for further revealing the role of the LHb in the circadian rhythm system.
